# An oceanic perspective on Greenland’s recent freshwater discharge since 1850

**DOI:** 10.1038/s41598-019-53723-z

**Published:** 2019-11-27

**Authors:** Kerstin Perner, Matthias Moros, Odd Helge Otterå, Thomas Blanz, Ralph R. Schneider, Eystein Jansen

**Affiliations:** 1 Department of Marine Geology, Leibniz Institute for Baltic Sea Research, See Str. 15, 18119 Rostock, Germany; 2 Department of Earth Science, University of Bergen and Bjerknes Centre for Climate Research, Allégaten 41, 5055 Bergen, Norway; 3 NORCE Norwegian Research Centre AS, Bjerknes Centre for Climate Research, Jahnebakken 5, 5007 Bergen, Norway; 4 Institute of Geosciences, Kiel University, Ludwig-Meyn-Straße 10, 24118 Kiel, Germany

**Keywords:** Palaeoceanography, Environmental impact

## Abstract

Instrumental data evidence an accelerating freshwater release from Arctic sea ice export and the Greenland Ice Sheet over the past three decades causing cooling and freshening in the subpolar North Atlantic region. However, evaluating the observed acceleration on a historical oceanic and climatic perspective remains challenging given the short available instrumental time series. Here we provide a marine perspective on the freshwater releases to the ocean since 1850 as reflected in the northern limb of the Subpolar Gyre. Our reconstructions suggest that the recent acceleration tracks back to the 1940s/50s and is unprecedented since 1850. The melting, initiated by the 1920s natural rise in solar irradiance, accelerated in response to a combined effect of natural and anthropogenic forcing factors. We find that Greenland’s freshwater discharge has contributed to a nutrient-driven fertilization of the upper ocean and consequently increased the marine primary productivity since the 1940s/50s.

## Introduction

The alarming 21^st^ century acceleration in the melting of Arctic sea ice and the Greenland Ice Sheet (GrIS)^1,2^ leads to a gradual freshening of the North Atlantic’s surface water layer^3–5^, affects ocean circulation^6,7^ and contributes to the rising global mean sea level^8^. Evaluating the observed acceleration on a historical perspective and identifying the trigger mechanisms (natural vs anthropogenic) is challenging as instrumental data cover, at most, the last few decades. A mid-19^th^ century perspective on freshwater release from the GrIS and drift/sea ice is of importance as the climate system has seen significant natural changes irrespective of any anthropogenic impact since the end of the Little Ice Age (LIA) around 1850^9^.

Drift/sea ice and freshwater from the Arctic Ocean and the eastern GrIS flow within the East Greenland Current (EGC) into the subpolar North Atlantic, of which up to 60% will reach the Labrador Sea^10^. Freshwaters potentially modulate Labrador Sea deep-water formation and thus influence the Atlantic Meridional Overturning Circulation (AMOC)^7^. As a result of the ocean’s surface freshening, a 15% weakening of the AMOC occurred since the mid-20^th^ century^11^. There is mounting evidence that the freshwater released from drift/sea ice and GrIS delivers high amounts of nutrients (dissolved organic carbon, P, Fe and N), potentially fertilizing the ocean by enhancing marine primary production, which may in turn also influence the marine food web and carbon cycle (refs ^12–15^ and references therein).

**Figure 1:**
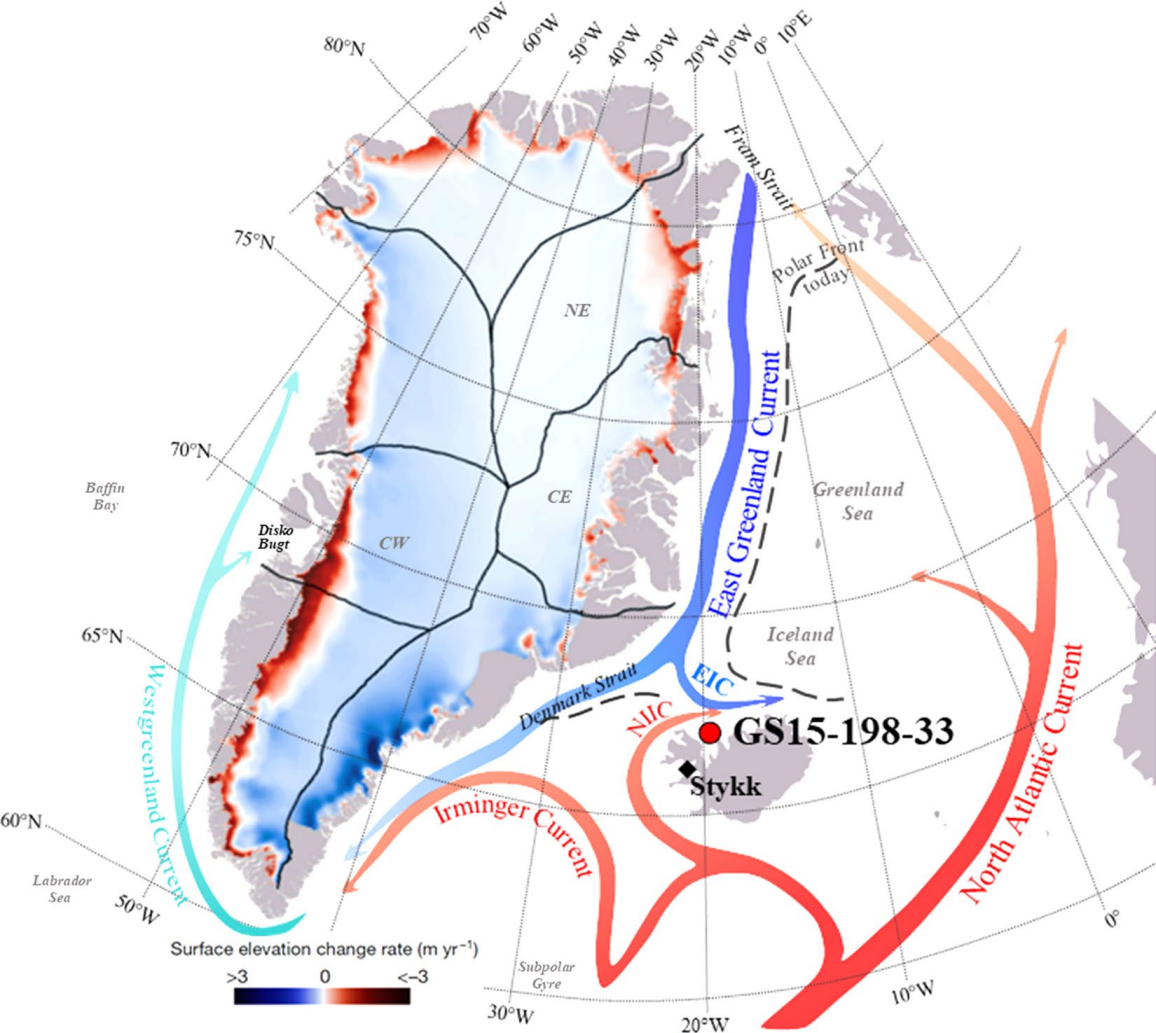
Location of sediment core GS15-198-33 on the North Icelandic shelf and regional ocean circulation in the subpolar North Atlantic. The North Atlantic Current (NAC) transports warm/saline waters northwards via eastern Fram Strait into the Arctic Ocean (red arrow). Part of the NAC flows westwards as the Irminger Current and North Icelandic Irminger Current (NIIC). Via western Fram Strait, cold/fresh surface waters (blue arrow) enter the subpolar North Atlantic and flow within the East Greenland Current and East Icelandic Current (EIC) southwards and form the northern limb of the Subpolar Gyre. The Polar Front (PF) reflects the approximate maximum extent of sea ice. We show surface mass balance changes of the Greenland Ice Sheet (GrIS) since 1900 obtained from ref. ^5^, which enter as meltwater releases the adjacent ocean. Sub-regions of the GrIS from ref. ^5^ present the respective drainage area as follows: NE – northeast, CE – central east, CW – central west. Stykk = Stykkishólmur weather station.

Scarce historical observations (visual detection from land or ships) provide information on sea ice extent in the past^16,17^, which marks the location of the Polar Front in the sub-polar North Atlantic region. The Polar Front separates cold and fresh waters from the EGC from warm and saline waters transported northwards with the North Atlantic Current (NAC) and the Irminger Current (IC). Presently the Polar Front is located further north seaward of the shelf in the Iceland Sea (Fig. 1). Southward shifts of the Polar Front caused by outbursts of cold and fresh waters from the Arctic Ocean, were commonly seen during the Great Salinity Anomalies (GSA’s)^18^ from the 1960–1990s. These events lead to widespread changes in drift/sea ice extent and freshwater input to the subpolar North Atlantic Ocean (refs ^17,19^ and references therein).

Here we present a marine record from the western North Icelandic shelf that provides a decadal-scale perspective (since 1850) on freshwater releases from melting Arctic Ocean drift/sea ice and the GrIS entrained into the EGC that has hitherto not been available. Core GS15-198-33 (site located at 66°37.5′N–20°51.2′W; Fig. 1) was collected from the Húnaflóaáll area, a north-south orientated depression, at 360 m water depth (Supplementary Note 1). We use a combined approach of proxy-based (alkenone and planktic foraminifera) and observational data (Supplementary Notes 2–4). The EGC influences the surface waters of the region, while subsurface water properties are closely linked to the inflow of Atlantic waters^20^ (Fig. 1, Supplementary Fig. 1). Therefore, our study offers an extended perspective, beyond the instrumental data period, on important environmental responses (e.g., ocean circulation, Polar Front movements and marine phytoplankton primary production) to changes in the climatic background conditions and freshwater release from the GrIS and drift/sea ice during the 19^th^ and 20^th^ centuries.

## Results

### Freshwater release and primary productivity on the North Icelandic shelf since 1850

**Figure 2:**
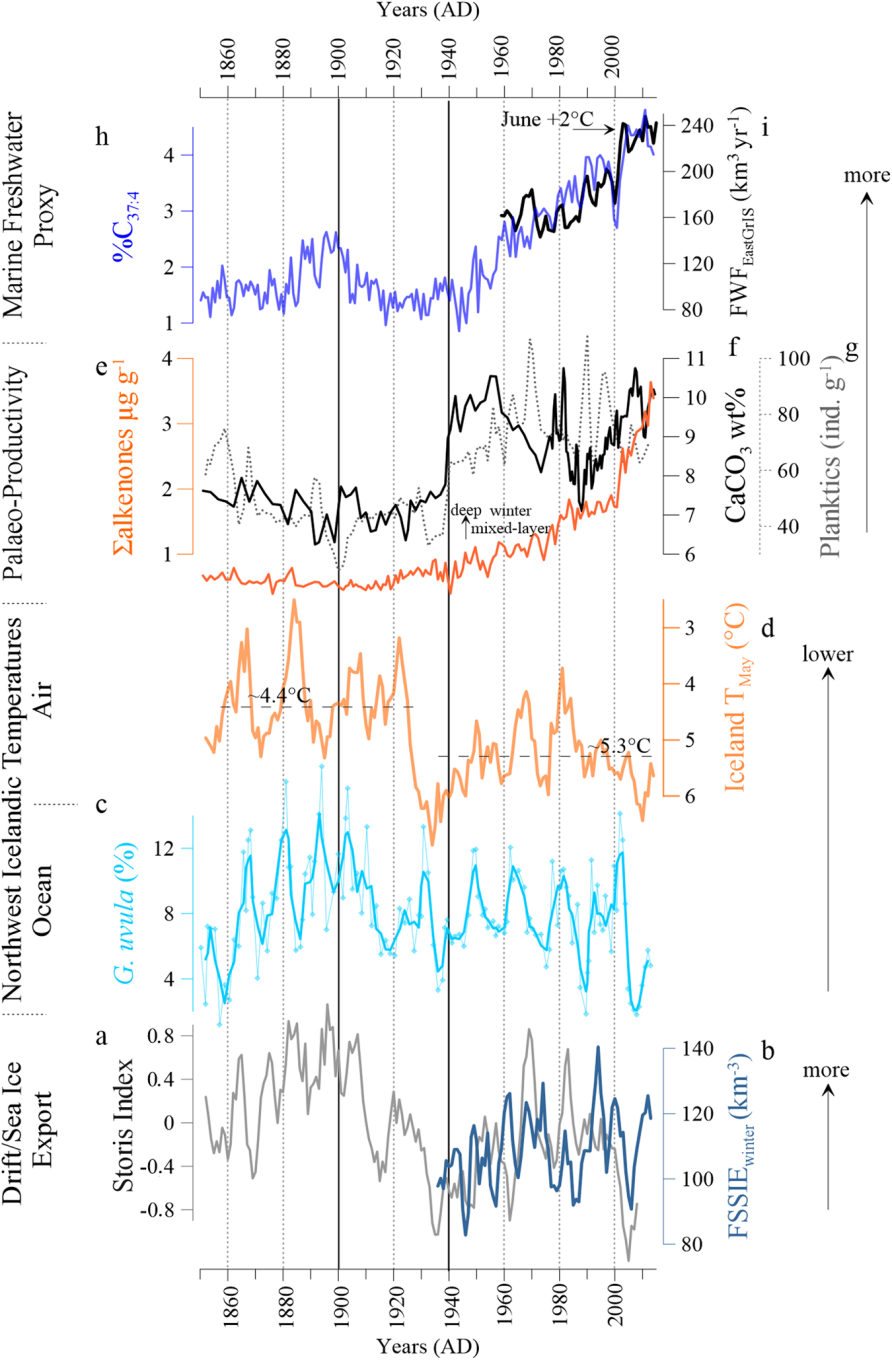
Changes in the southward influence of drift/sea ice and freshwaters since 1850. (**a**) Observational/historical drift/sea ice occurrence on the East Greenland shelf^22^, (**b)** western Fram Strait satellite –based reconstruction of the winter sea-ice export^21^, (**c)** abundance (%) of the cold and fresh water indicator *G. uvula* – thick blue line is the 3 point running mean, (**d)** West Icelandic air temperature from Stykkishólmur – please note the inverse axis scaling, **e** calcium carbonate content (CaCO_3_) **(f)**, the flux of planktic foraminifera g^−1^ (dashed line) (**g)** Total sum (Σ) of all alkenones. (**h)** Total abundance (%) of the freshwater alkenone C_37:4_, (**i)** Annual Freshwater Flux (FWF) to the Greenland Sea (GrSea)^3^.

**Figure 3:**
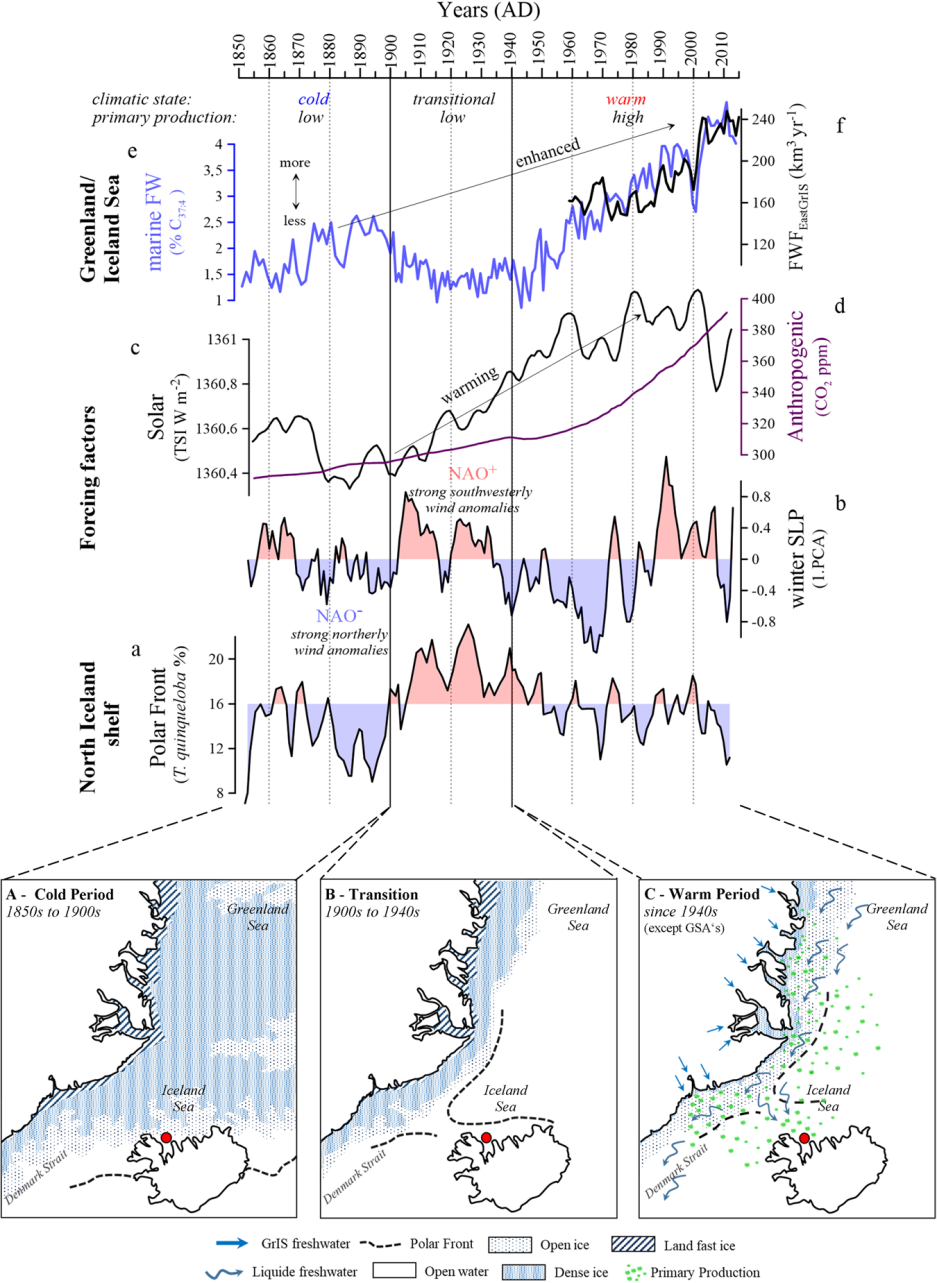
Oceanic response to drift/sea ice retreat. (**a)** Changes in marine surface freshwater occurrence. (**b)** Annual freshwater Flux (FWF) to the Greenland Sea as presented in ref. ^3^. (**c**) Solar irradiance as shown in ref. ^23^. (**d)** Atmospheric CO_2_ concentration obtained from https://data.giss.nasa.gov/modelforce/ghgases/Fig.1A.ext.txt. (**e**) The leading Principal Component (PC) from an EOF analysis of the winter Sea Level Pressure (SLP) for the region 90°W to 40°E and 20°N to 70°N calculated from the HADSLP 2014 data set^24^, reflecting the North Atlantic Oscillation (NAO) pattern. **(f)** Abundance (%) of Polar Front indicator *T. quinqueloba*.

An investigation of the frontal zone area of the North Icelandic shelf reveals multi-decadal fluctuations in regional oceanic conditions (Fig. 2c,e,g) and atmospheric temperatures (Fig. 2d) since 1850. This variability is concurrent with the 38 years long drift/sea ice export history from western Fram Strait^21^ and the reconstructed Storis Index from southeast Greenland^22^ (Fig. 2a,b). Apparent co-variability in the North Icelandic ocean and air temperatures and western Fram Strait sea ice export during the 20th century^21,22^ suggests that regional air and surface ocean climate vary here in response to the occurrence of drift/sea ice in winter and spring. The abundance of %C_37:4_ and the recent freshwater flux record (FWF – combines meltwater from the eastern GrIS and Fram Strait drift/sea ice) to the Greenland Sea^3^ co-vary closely during the last five decades (Fig. 2h–i). This enables us to extend the available Greenland Sea FWF record^3^ qualitatively beyond the instrumental data period. Changes in climatic forcing factors (Table 1), i.e. solar irradiance^23^ (Fig. 3c), anthropogenic forcing (Fig. 3d), atmospheric circulation^24^ (NAO, Fig. 3b) and oceanic forcing (Atlantic Multidecadal Oscillation – AMO; see Table 1 and Supplementary Fig. 5d), are likely to control large parts of the overall variability and trends in regional freshwater and primary production. In the following, we examine the link between various forcing mechanisms and the resulting oceanic responses since 1850, using the following tripartite division: (1) 1850s to 1900s, (2) 1900 to 1940s/50s, and (3) 1940s/50s to the present.

**Table 1:** Summary of changes in oceanic/climatic conditions on the North Icelandic Shelf and key climate forcing factors for the Atlantic region since 1850.

	Proxy	1850 to 1900	1900s to 1940s/50s	1940s/50s to present		
				1940s/50s to 1980s	1980s to 2000	since 2000
regional North Icelandic conditions	Drift/ Sea Ice occurrence	high	low	increasing	decreasing	absent
	Polar Front	weak	strong	weak	weak	weak
	Summer to Winter Air Temp. gradient	high	decreasing	increasing	decreasing	decreasing
	GrIS freshwater supply	low	low	increasing	increased	increasing
Forcing factors	NAO Index	negative phase	positive phase	negative phase	positive phase	neagtive phase
	AMO Index	positive phase	negative phase	positive phase	negative phase	positive phase
	SPG-AMOC	strong	strong	weakening	weak	strong
	Volcanic activity	weak	weak	increased	increased	weak
	Solar Irradiance	weak	increasing	increasing	high	decreasing

#### Late LIA conditions (1850s to 1900s)

A constant supply of drift/ sea ice^25^ traveling through the EGC and East Icelandic Current (EIC; Fig. 1) to the North Icelandic shelf (Fig. 2a, Table 1) produce relatively cold ocean^26^ (Fig. 2c) and spring air temperatures in the wider study area (Fig. 2d). Concomitantly, we record low CaCO_3_ content and planktic foraminifera flux (<80 ind. g^−1^) that implicate a shallow/thin winter mixed-layer and low primary production (Fig. 2f,g). Low freshwater (%C_37:4_ <2.5%, Fig. 2h) and Σ alkenone abundance (<1 µg g^−1^, Fig. 2e) support the assumption of a weak surface primary production which is presumably limited to the spring months. This, combined with the low abundance of the front indicator *T. quinqueloba*^26^ (Fig. 3a), suggests a weak Polar Front influence on the North Icelandic shelf. The drift/sea ice that regularly reached the North Icelandic shelf primarily controlled oceanic conditions, while the Polar Front lingered south of Iceland^16,27^ (Fig. 3A). In winter, the prevalence of strong northerly winds (negative NAO; Fig. 3b) favoured a southward extension of the drift/sea ice (Fig. 3A) in the subpolar North Atlantic region. The weak solar irradiance^23^ at this time (Fig. 3c) likely hampered quick melting of the early spring drift/sea ice and consequently prevented excessive freshwater release to the surface ocean layer. This allowed the development of a stable stratification, which limited vertical mixing and heat loss to the atmosphere and prohibited shoaling of the winter mixed-layer during spring. As shown by our data (Figs. 2, 3A), the abundant drift/sea ice occurrence in spring restricted nutrient availability to the surface layer and thus hindered excessive primary production (Fig. 2f,g).

#### Changing climatic background conditions (1900 to 1940s/50s)

Overall, low freshwater occurrence (Fig. 2h) and weak primary production (Fig. 2f,g) still prevailed on the North Icelandic shelf during the early-20^th^ century. As the atmospheric pressure pattern shifted towards a more positive NAO phase, with strong southwesterly wind anomalies in winter (Fig. 3b), less drift/sea ice reached the shelf region, which in turn initiated a northward migration of the Polar Front from its late-LIA position south of Iceland. The prominent rise in *T. quinqueloba* (Fig. 3a) illustrates the presence of the Polar Front on the North Icelandic shelf during spring, when there was no sea ice over the site anymore (Fig. 3B). In the 1900s to early-1920s, relatively large summer to winter temperature differences of about 14 °C occur (Table 1; Supplementary Fig. 5a). In the ocean, surface waters warm in spring (Fig. 2c) concurrent with decreasing drift/sea ice occurrence^16^ (Table 1; Fig. 2a and Supplementary Fig. 5h), while the winter mixed-layer remained relatively shallow (Fig. 2f,g).

The 1920s gradual increase in total solar irradiance (Fig. 3c) warmed the northern hemisphere and marked the transition from a ‘cold’ to a ‘warm’ climatic state. This transition parallels a lull in volcanic activity that likely contributed to a rising effective solar irradiance^28^ (see Table 1). Concurrently, the atmospheric CO_2_ concentrations showed a weak positive trend (Fig. 3d). The air temperature increased markedly in the western Iceland area (Fig. 2d) as well as on the southeastern Greenland shelf^29^, and the summer to winter air temperature difference decreased to about 9 °C (Supplementary Fig. 5a). Simultaneously, drift/sea ice occurrence^16^ (Fig. 2a) reached an all-time 20^th^ century low and became nearly absent on the shelf during the 1930s (Table 1, Supplementary Fig. 5h). These conditions correspond to an overall atmospheric (Fig. 2d) and oceanic (Supplementary Fig. 5d) warming during that time^22,29^. The increasing inflow of warm Atlantic Water into the subpolar North Atlantic likely contributed to atmospheric warming of the entire Arctic^29^ and initiated from the early 1920s a stepwise subsurface warming on the North Icelandic shelf^30^ (Supplementary Fig. 3e). Despite the return to a negative NAO phase, and subsequent strong northerly wind anomalies in winter (Fig. 3b), the oceanic gradient weakened on the shelf in the mid-1930s, as the absence of drift/sea ice could not possibly cause regional cooling of air and surface ocean temperatures compared to the 1850 to 1900 interval. Nevertheless, our reconstructions reveal that the increase in solar irradiance and shift to a positive NAO phase likely triggered the observed changes in freshwater abundance and primary production on the North Icelandic shelf for the 1900 to 1940s/50s interval. The pronounced minimum in our C_37:4_ record (Fig. 2h) suggests that the freshwater influence is confined to the west of the Polar Front on the East Greenland shelf during this period.

#### An accelerated freshwater release to the ocean (from 1940s/50s to the present)

A permanently boosted primary production (Fig. 2f,g) accompanied an accelerated surface water freshening (Fig. 2h) and a reduced Polar Front influence (Figs. 3a,c) since the 1940s/50s on the shelf region. The climatic background conditions, i.e. high solar irradiance^23^ (Fig. 3c), prevailed now in a ‘warm state’ (Table 1) and radiative forcing from anthropogenic greenhouse gas emissions increased continuously (Fig. 3d). We attribute the accelerated freshwater release to enhanced melting of drift/sea ice and an amplified freshwater flux from the Arctic Ocean (Fig. 3c), concurrent with an increased meltwater runoff from the eastern part of the GrIS^3^ (Fig. 3f). This is in accordance with reports of a permanently reduced surface mass balance of the GrIS since the 1950s^31^. Our primary production reconstruction demonstrates prominent decadal variability since the 1940s/50s (Fig. 2e,f), while our freshwater proxy data illustrate the increased accumulation of freshwater in the upper ocean (Fig. 2h,i). The primary production’s decadal variability likely resulted from various forcing factors acting on a local to regional scale that finally lead to enhanced vertical mixing on the North Icelandic shelf.

In response to the 1940s/50s high spring air temperatures (Fig. 2d), almost absent drift/sea ice^16^ (Supplementary Fig. 5h) and warm subsurface waters (Supplementary Fig. 3e), a pronounced, thermal-driven, stratification developed on the North Icelandic shelf. During this time, a weak Polar Front influence (Fig. 3c) prevailed in consequence of the weakened oceanic gradient, i.e. warm atmospheric and oceanic temperatures. These conditions led to the gradual formation of a deep and stable winter mixed-layer and allowed a rapid shoaling in spring. This initiated a prominent boost in primary production in the 1940s/50s by enhancing the coccolith (Σ alkenones >1 µg g^−1^, Fig. 2e) and planktic foraminifera blooms (Fig. 2g). The decline in primary production in the 1960s to late-1970s (the time of the GSA events), on the other hand, did not occur solely in response to thermal-driven winter mixed-layer depth changes. It also corresponded to an increased influence of drift/sea ice and the subsequent freshwater release to the surface water layer, as the Polar Front migrated over the North Icelandic shelf and prevailed further south of our core site near its LIA position (Fig. 3a). The intensified drift/sea ice export through the western Fram Strait^16,21^ (Fig. 2a,b) was favoured by a negative NAO phase, with strong northerly wind anomalies (Fig. 3c, Supplementary Fig. 4a). This return to more abundant drift/sea ice (Supplementary Fig. 5h), as opposed to the preceding drift/sea ice minimum in the 1930s-1950s, led to renewed cooling of the air and surface water temperatures in the region in the 1960s to 1970s (Fig. 2c,d). Although air temperatures dropped, the summer to winter temperature difference remained about 2 °C higher compared to the preceding negative NAO phase from 1880 to 1900 (Supplementary Fig. 5a). Such a seasonal air temperature difference between a ‘cold’ and a ‘warm’ climate state seems to be sufficient to trigger an earlier annual melting onset of drift/sea ice, producing notably freshwaters, as evidenced by our data (Fig. 3e). Simultaneous to widespread oceanic cooling across the North Atlantic basin (Supplementary Fig. 5d) and a slowdown of the ocean circulation^11^ (Supplementary Fig. 5f) during the 1960s to late-1970s, cooler intermediate waters reached our study area^30^ (Supplementary Fig. 3e). In contrast to the preceding 1940 to 1960 warm period, a thicker freshwater lid in the 1960s to late-1970s likely restricted any rapid shoaling of the winter mixed-layer and led to a temporally reduced, coccolith-driven, surface primary production (Fig. 2f). From the late-1970s to mid-1980s, surface (coccolith) primary production recovered (Fig. 2f), which occurred together with a brief cooling in air temperatures as well as in surface waters (Fig. 2c,d). This accompanies a steep rise in subsurface temperatures on the shelf^30^ (Supplementary Fig. 3e). We suggest that the return to a thermal-driven stratification caused the deepening of the winter mixed-layer and its rapid shoaling in spring that produced the peak in (coccolith) primary production (Fig. 2f).

In the 1980s to 2000s, the NAO shifted into a pronounced positive phase (Fig. 3b) and the return to strong southwesterly wind anomalies presumably favoured an enhanced accumulation of freshwater on the North Icelandic shelf. The constant weak Polar Front influence (Fig. 3a) seems to result from the markedly freshened surface waters. Simultaneously, the seasonal air temperature difference decreased roughly by another 4 °C (Supplementary Fig. 5a), and was mainly driven by a rise in spring temperatures (Fig. 2d). This consequently promoted a faster and prolonged melting of drift/sea ice. Combined with a predominant export of younger and thinner drift/sea ice from the Arctic Ocean (refs.^1,2^ and references therein), the freshwater release to the upper ocean layer intensified markedly (Fig. 3e,f). As a consequence of this, stable stratification developed on the shelf, which prevented a shoaling of the winter mixed-layer and thus reduced the surface primary production.

Since the 2000s, the seasonal temperature gradient decreased by a further 2 °C (Supplementary Fig. 5a) compared to the preceding interval. Northwestern Icelandic spring temperatures rose by about 1 °C (Fig. 2d) and June temperatures increased by as much as 2 °C (Fig. 3), despite the fact that solar irradiance decreased during this time (Fig. 3c). Substantially diminished drift/sea ice concentrations along the East Greenland shelf was observed by the year 2000 and the Polar Front retreated northward into the Iceland Sea (Fig. 3C). The gradual disappearance of seasonal ice increased the heat flux to the atmosphere through enhanced upper ocean convection, which is typically limited to the depth of the mixed-layer^32,33^. Concurrently, surface freshening accelerated on the North Icelandic shelf (Fig. 2h,i), while high atmospheric and oceanic temperatures triggered enhanced surface and subsurface melting of the GrIS^34,35^ and caused an excessive release of melt- and freshwater to the marine environment^3,36^ (Supplementary Fig. 6). Recent observational studies suggest that rising carbon emissions^37^ (Fig. 3d), increased water vapour (aerosol load)^38^ and redundant deposition of ‘black carbon’ on the GrIS and sea ice^39^ could act as crucial factors for enhancing the melting. Other recent works suggest that the GrIS and its peripheral glaciers may exceed a tipping point, where surface mass loss and surface runoff outpace the re-growth and thus destabilize the GrIS^40,41^. It appears that anthropogenic forcing has accelerated this melting process of the drift/sea ice and the GrIS since the early 2000s via feedback processes (i.e. albedo reduction)^42^, while the influence of natural (solar and oceanic forcing) drivers seem to have diminished.

## Discussion

### Acceleration of ocean freshening since the 1940s/50s – a regional perspective

During the 1940s/50s, the accelerated freshening of the northern Subpolar Gyre limb was likely the result of the natural change in the climatic background conditions, i.e. solar, atmospheric and oceanic forcing, since the end of the LIA (Fig. 3). Since the 1920s atmospheric warming substantially reduced the sub-Arctic and Arctic seasonal temperature gradient (Supplementary Fig. 5a), and as a result the drift/sea ice and the GrIS became more vulnerable. In addition, the shift to a more positive NAO (Fig. 3e) led to more moist air masses being diverted poleward and thus strengthened the meridional northward heat transport. The associated atmospheric warming led, in turn, to a prolonged melting season. Starting in the late-1920s, a substantial warming took place in the North Atlantic as reflected by the positive AMO Index (Supplementary Fig. 5d). The combination of strong atmospheric and oceanic forcing likely contributed to the rise in GrIS meltwater runoff in the 1930s^32^, and triggered enhanced drift/sea ice melting that eventually caused freshwater accumulation in the Arctic Ocean^43^. This strong early 20^th^ century Arctic warming happened at a time when the levels of carbon emissions were much lower compared to recent decades (Fig. 3d), which suggests that anthropogenic forcing played a minor role. The subsequent mid-1930s shift to a negative NAO phase (Fig. 3b) favoured an accelerated release of freshwater and drift/sea ice from the Arctic Ocean via the western Fram Strait. This accelerated Arctic freshwater release, which merged with the GrIS runoff within the EGC, reached the North Icelandic shelf in the 1940s/50s (Fig. 2c–i). We propose that since that time this freshwater forcing has exerted a prominent control on the regional environmental conditions, including the Greenland and Iceland Sea. Importantly, the freshwater transport in the EGC through the Denmark Strait (Fig. 1) is part of the northern limb of the Subpolar Gyre. Therefore, we postulate that since the 1940s/50s the Labrador Sea has received larger amounts of freshwater from the EGC, as compared to the background climate conditions during the LIA. Our findings highlight that the 1960s–1990s ‘Great Salinity Anomalies’^18^ and the recent (since 2000) rise in GrIS freshwater flux^3^ should be considered as multi-decadal climate variability against the backdrop of this longer-term freshening trend. The 1960s to late-1980s pronounced negative NAO phase certainly facilitated strong freshwater release from the Arctic Ocean. Presumably, the shift from a ‘cold’ to a ‘warm’ climatic state allowed distinct fingerprints of these outflows on the simulated AMOC^11^ and associated Subpolar Gyre (ref. ^10^ and references therein) variability to be examined. In association with the 1990s shift to a positive NAO, the freshwater outflow from the Arctic Ocean was reduced^44^, which allowed unprecedented amounts of freshwater to accumulate in the Beaufort Gyre since the start of the 21^st^ century^45,46^. However, instead of a slowdown of the subpolar North Atlantic’s surface water freshening since the 1990s, we document in accordance with recent oceanographic studies^3,6,47^ a continuation. The 1940s/50s initiated melting of the GrIS evolved during the 2000s as the major freshwater source to the Subpolar Gyre’s northern limb and thus to the subpolar North Atlantic. Despite the negative trend in solar irradiance forcing since the 2000s (Fig. 3c), the northern hemisphere has experienced significant climatic warming (Supplementary Fig. 5e) and ice sheet melting, and drift/sea ice retreat continued at an unprecedented rate similar to carbon emissions (Fig. 3d) and the global sea-level rise^8^. This suggests anthropogenic forcing as the main driver for the recently observed changes in the freshwater fluxes.

### Freshwater controlled acceleration of marine primary production since the 1940s/50s

Recent oceanographic studies reveal that the freshwater flux from the GrIS and its peripheral glaciers not only occurs as surface runoff, but also as submarine discharge^15,48^. The surface and subsurface freshwater releases from the GrIS act as dynamic nutrient sources to the ocean and thus significantly influence marine primary production^48,49^, a factor that will become even more important if climate warming continues. The co-variance between our freshwater record and the GrIS FWF record^3^ demonstrates the close coupling of surface marine production and nutrient release from the GrIS since the 1940s/50s. Our findings are in line with recent studies on sub-glacial meltwater discharge^48^ and nutrient release from meltwaters^12^ from East and West Greenland^13,49^. A similar GrIS-melt driven fertilizing effect of surface waters, as recorded on the North Icelandic shelf, appears also to take place in the Disko Bugt area, West Greenland^36^ (Supplementary Fig. 6). Furthermore, downstream in the western limb of the Subpolar Gyre, an area influenced by the cold and drift/sea ice carrying Labrador Current, recent studies reveal increased primary production driven by reduced ice occurrence^50^. These findings point to the so far under communicated, but import role of meltwaters from drift/sea ice and the GrIS as dynamic sources of nutrients for the oceans. This has important implications for the role of the ocean as a carbon sink to the deep-ocean and nutrient re-distributer.

### Summary

Acceleration in freshwater release from the GrIS and drift/sea ice of the last 30 years, as documented in instrumental data, is unprecedented since 1850. Our combined analyses of historical and proxy data provide evidence that this surface water freshening in the subpolar North Atlantic started already back in the 1940s/50s, and is part of a longer-term process. Starting in the 1900s, the stepwise overlapping of natural forcing factors, such as the 1920s atmospheric warming and shift to a positive NAO followed by the oceanic warming in the 1930s, triggered the acceleration in freshwater discharge to the upper ocean in the 1940s/50s. Since the 1960s, the anthropogenic forcing has added to the natural forcing factors that lead to melting of the GrIS. As a side effect of this, nutrient-rich freshwater have contributed to enhanced fertilization of the ocean by increasing marine primary production. Our results provide a new perspective on the ocean freshening since 1850, and reveal that changes in freshwater release recorded in observations covering the last 30 years are rather small in amplitude on this longer-term perspective.

## Methods

### Sediment core collection

The marine sediment core GS15-198-33 was collected by the *R/V* ‘G.O. Sars’ in 2015 at 66°37.53′N, 20°51.16′W from 361 m water depth offshore western North Iceland. Core GS15-198-33 has been collected from a well-known high accumulation area on the shelf that was previously sampled in 1999 by the *R/V* ‘Marion Dufresne’ (core MD99-2269)^25^. The sediment core was sampled continuously at 1 cm resolution (1 cm thick sediment slices).

### Radionuclide measurements

Radionuclide analyses of ^210^Pb, ^137^Cs, and ^241^Am were carried out by gamma spectrometry with a Ge-well detector (GCW4021-7500SL-RDC-6-ULB) and processed with GENIE 2000 software (Canberra Industries Inc., USA). Counting statistics were better than 15% for ^210^Pb and ^137^Cs and better than 20% for ^241^Am activity. The radionuclide activities were calculated using standard reference materials (decay corrected): IAEA-447 (^137^Cs, ^210^Pb, ^226^Ra) and IAEA-385 (^241^Am). The following nuclides and energies were used for quantification of isotopes: ^210^Pb: 46.5 keV, ^226^Ra: 295 keV and 351 keV, ^241^Am: 59.5 keV and ^137^Cs: 661 keV.

### Bulk sediment analyses

For mercury measurements, we used a DMA-80 analyzer from MLS Company. Data were calibrated against CRM (BCR) 142R certified reference material and SRM 2709 soil standard using 5 concentration steps covering a range from 5 to 500 ng Hg. Sample weights were 100 mg. The CaCO_3_ content was calculated using the sediments total inorganic carbon content (TIC). The TIC was measured using 100 mg of freeze-dried sediment that was diluted with 40% H3PO4 and incinerated at 1200 °C on a Multi EA4000 from Analytikjena.

### Planktic foraminifera counts

We determined planktic foraminifera down to species level in the >63 µm using a stereomicroscope. We recorded six planktic foraminifera species of which *Turborotalita quinqueloba*, *Globigerinita uvula*, *Neogloboquadrina pachyderma*, and *Globigerinita glutinata* occur in high abundance (>6%), while *Globigerina bulloides* and *Globorotalia scitula* occur only sporadically.

### Preparation and analyses of alkenones

Homogenized sediments samples were analyzed for alkenones out at the Institute of Geosciences Biomarker Laboratory, Kiel University. Long-chained alkenones (C_37_) were extracted from homogenized, 2 to 3 grams of bulk sediment, using an Accelerated Solvent Extractor (Dionex ASE-200) with a mixture of 9:1 (v/v) of dichloromethane:methanol (DCM:MeOH) at 100 °C and 100 bar N_2_ (g) pressure for 20 minutes. Extracts were cooled at *c*. −20 °C and dried by vacuum rotary evaporation at 20 °C and 65 mbar. We used a multi-dimensional, double gas column chromatography (MD-GC) set up with two Agilent 6890 gas chromatographs for identification and quantification of C_37:2_ and C_37:3_ ketones^51^. Quantification of the individual compounds was achieved with the addition of an internal standard prior to extraction (cholestane [C_37_H_48_] and hexatriacontane [C_36_H_74_]. The relative proportions were obtained using the peak areas of the two different compounds. The UK′_37_ index was calculated using the equation^52^: UK′_37_ = (C_37:2_)/(C_37:2_ + C_37:3_). The proportion of tetra-unsaturated C_37_ ketones relative to the sum of alkenones (%C_37:4_) serves as an indicator of changes in freshwater release, via meltwater discharge, from the GrIS^53,54^.

### Empirical orthogonal function analyses

Standard linear regression has been used to calculate the regression maps in Supplementary Fig. 4, whereas the empirical orthogonal functions have been calculated using standard singular-value decomposition analysis. We have used winter (December-January-February) sea level pressure data from the HadSLP2 data set^24^ over the Atlantic sector (90°W–40°E; 20°N–70°N). HadSLP2 data is provided by the NOAA/OAR/ESRL PSD, Boulder, Colorado, USA, from their Web site at https://www.esrl.noaa.gov/psd/.

## Supplementary information

Supplementary information

Supplementary Dataset 1

## Data Availability

The data presented within this manuscript are available as excel file.
