# Supplementary material for: An oceanic perspective on Greenland’s recent freshwater discharge since 1850
Source: Sci Rep. 2019 Nov 27;9:17680. doi: 10.1038/s41598-019-53723-z (PMC6881324; doi:10.1038/s41598-019-53723-z)
Supplement: Supplementary file 1 — Supplementary information [file 41598_2019_53723_MOESM1_ESM.docx]

Supplementary Information

**An oceanic perspective on Greenland’s recent freshwater discharge since 1850**

Kerstin Perner^1,2,3*^, Matthias Moros^1*^, Odd Helge Otterå^3^, Thomas Blanz^4^, Ralph R. Schneider^4^, and Eystein Jansen^2,3^

1Department of Marine Geology, Leibniz Institute for Baltic Sea Research, See Str. 15, 18119 Rostock, Germany

2Department of Earth Science, University of Bergen and Bjerknes Centre for Climate Research, Allégaten 41, 5055 Bergen, Norway

3 NORCE & Bjerknes Centre for Climate Research, Jahnebakken 5, 5007 Bergen, Norway

4Department of Geology, Institute of Geosciences, Kiel University, Ludwig-Meyn-Straße 10, 24118 Kiel, Germany

**Supplementary Note 1: Site selection, North Icelandic shelf.** The North Icelandic shelf is known for its high-resolution sedimentary archives^1^. Core GS15-198-33 (site located at 66°37.5’N-20°51.2’W; Supplementary Fig. 1) was collected from the Húnaflóaáll area, a north-south orientated depression, at 360 m water depth. The *RV Marion Dufresne* has previously sampled this site in 1999 (core MD99-2269). In situ hydrographic measurements, conducted during the cruise GS15-198 in late July 2015, show a strong vertical stratification (Supplementary Fig. 1). The East Icelandic Current (EIC) influenced surface waters (< 25 m) are fresh and well ventilated and underlain by more saline waters (25 to >200 m), the Subpolar Mode Waters (SPMW) that are carried within the North Icelandic Irminger Current (NIIC) along the North Icelandic shelf.

**
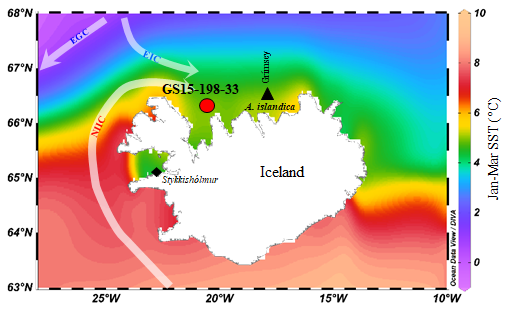

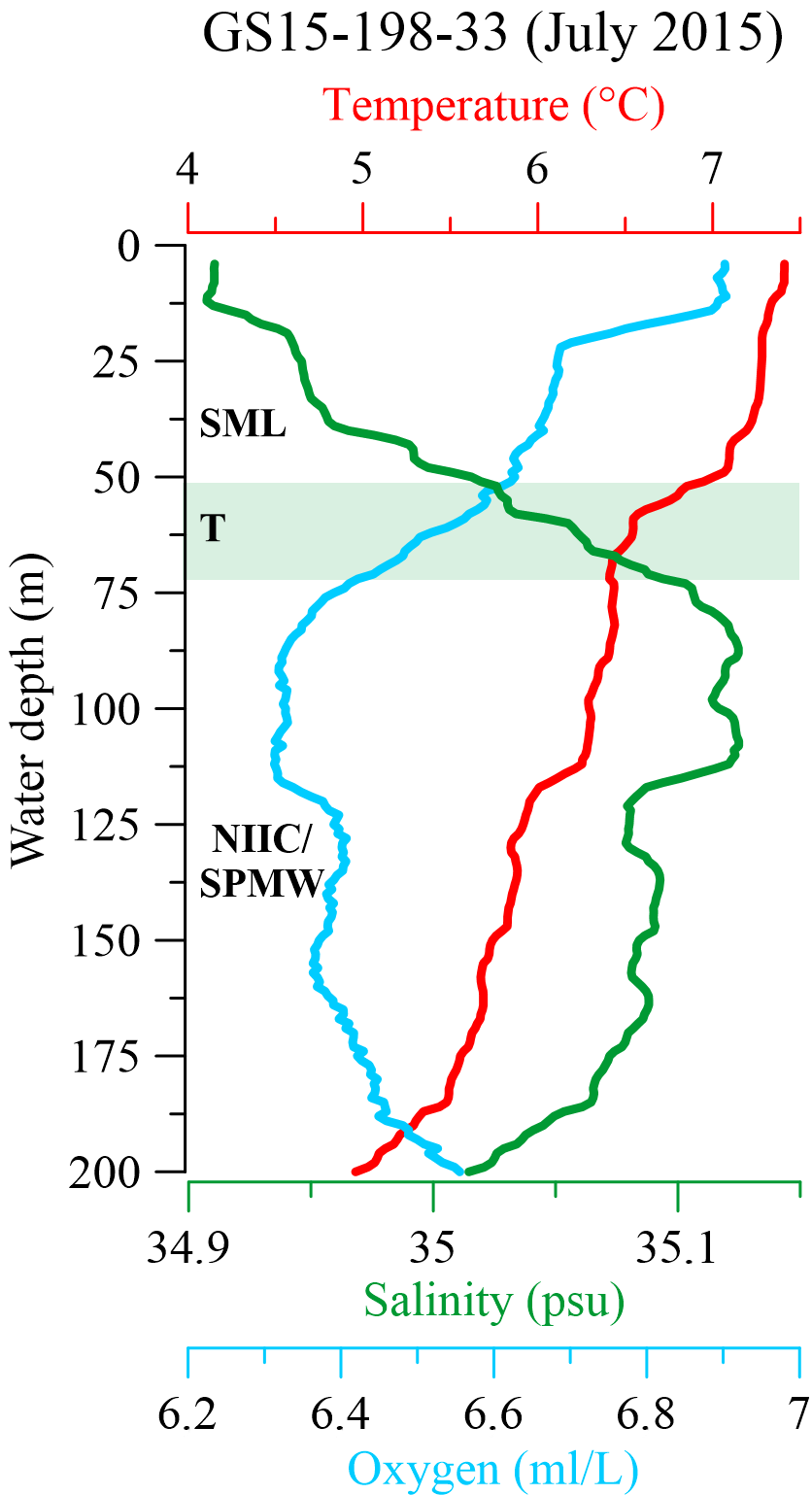
**

**Supplementary Fig. 1: Modern oceanic conditions on the North Icelandic shelf.** (*left*) Modelled winter (January to March) sea surface temperature (SST) obtained from the World Ocean Data Base 2013 using Ocean Data View^2^. Location of sediment core GS15-198-33 (red circle, this study) and collection site of *Arctica islandica* shells near Grímsey Island^3^ (black triangle) is shown. (*right*) Temperature (red), salinity (green) and oxygen (blue) profiles from site GS15-198-33 measured in July during the cruise.

**Supplementary Note 2: Sediments and chronology.** Core GS15-198-33 characterises fine-grained sediments that are primarily composed of silty clay. Comprehensive dating of recent marine sediments offers the combined application of radionuclide dating (natural Lead - ^210^Pb, artificial: Caesium - ^137^Cs, Americium - ^241^Am)^4,5^ and of the environmental pollution marker mercury (Hg)^6,7^ (see Supplementary Fig. 2). Atmospheric bomb testing from 1954 onwards introduced the ^137^Cs and ^241^Am into the marine environment, which provide a distinctive chronostratigraphic marker preserved within the sediment (refs. ^4,5^ and references therein). In particular, the activity of ^210^Pb_unsupp_ marks the approximate dating horizon^4^ of the year 1900 (Supplementary Fig. 2), which is found at 121 cm core depth. A first rise in the total Hg content above natural background values of < 20 µg kg^-1^ around the year 1900 marks the initiation of the anthropogenic influence^6,7^, which evolves distinctly from the 1960s onwards. Linear interpolation between the year of sampling (2015), our chronostratigraphic markers (^137^Cs, ^241^Am, ^210^Pb and Hg) and the end of the LIA (around 1850) reveals an average sedimentation rate of about 1 cm yr^-1^. Consequently, our proxy records resolve the last 165 years with an integrated resolution of 1 to 2 years per sample, as we used a 1 cm sample interval. This approach allows an age-depth model development that is not biased by reservoir age changes over time, which has been previously successfully shown from the Black^8^ and Baltic Sea^9^.


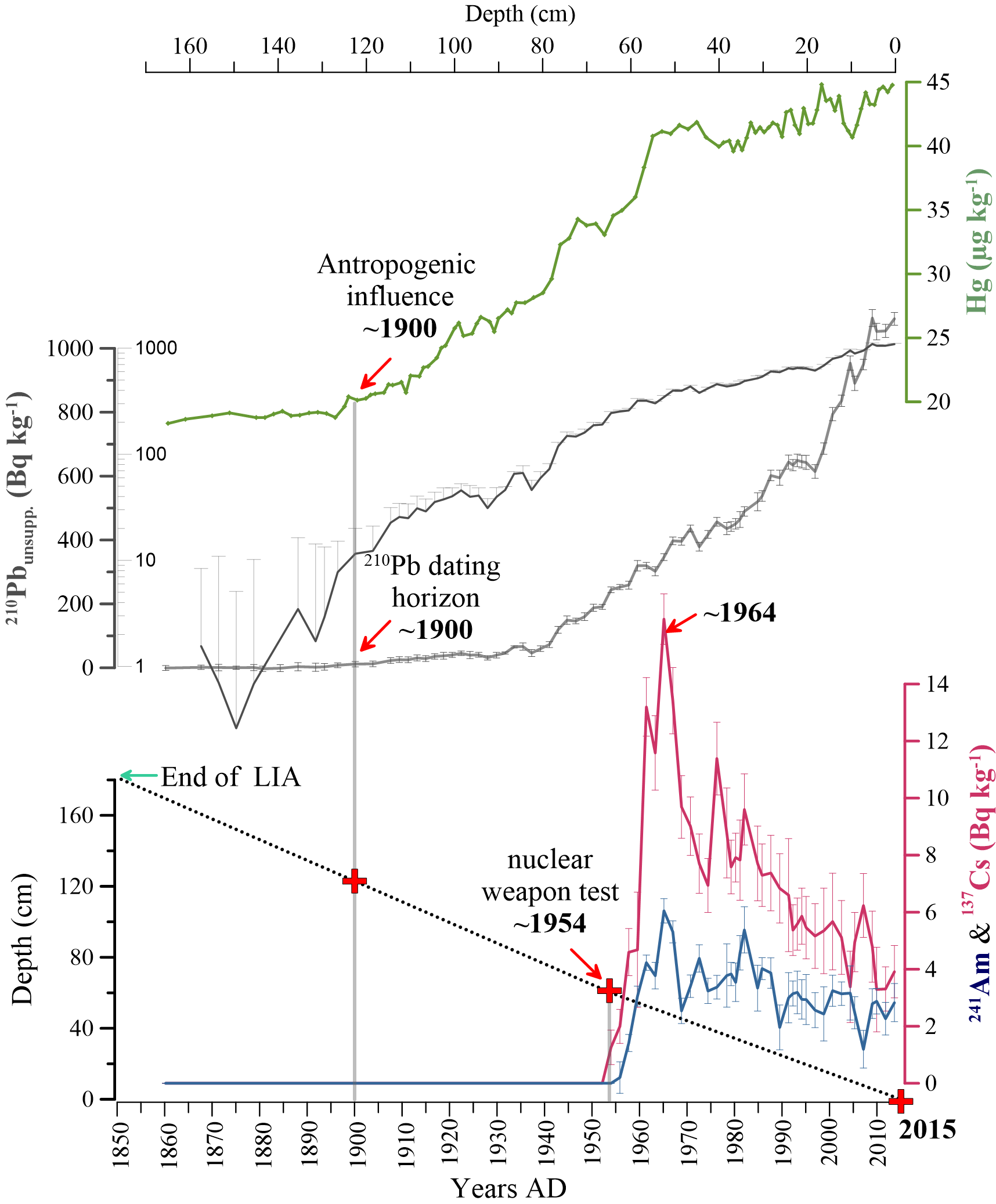


**Supplementary Fig. 2: Chronology of core GS15-198-33 since 1850.** The age-depth model is based on the combined information of chronological time markers obtained from the occurrence of natural and artificial radionuclides.

**Supplementary Note 3: Proxy validation.** An ideal tracer to record freshwater input to the ocean is the abundance of the C_37:4_ compound relative to the sum of all alkenones (in % of Σ alkenones), which is a proxy for the occurrence of cold and fresh polar waters^10^. We infer changes in surface and subsurface water primary production from the Σ alkenone record, the sediments CaCO_3_ content and the flux of planktic foraminifera (number of individuals per gram). Biogenic carbonate from coccoliths contributes to more than 50% of the CaCO_3_ total weight percent (wt%)^1^, while calcareous (planktic and benthic) foraminifera comprise roughly the rest.

The Σ alkenones, i.e. intensity of the coccolith bloom, serve as a proxy for surface water productivity in spring and early summer (Fig. 2c). Solar irradiance and freshwater abundance control the duration of the blooming period. Another factor that influences the magnitude of the spring bloom is the evolution of the winter mixed-layer, which determines the nutrient availability to the marine planktic organisms. Rapid shoaling of the deep winter mixed-layer enhances the near- to sub-surface primary production. Its relative depth (shallow/deep) varies in response to the occurrence of drift/sea ice, freshwater fluxes, Polar Front movements and atmospheric forcing^11^. The enhanced (low) marine primary production as inferred from the total CaCO_3_ wt% and planktic foraminifera flux, therefore, illustrates the formation of a deep (shallow) winter mixed-layer and its subsequent rapid (hampered) shoaling during spring on the North Icelandic shelf.

The polar planktic foraminifera *Globigerinita uvula* and *Turborotalita quinqueloba* thrive at the near-surface and within the mixed layer^12-16^ (Supplementary Fig. 3). The opportunistic *G. uvula* mirrors observed trends in spring (May) temperatures within the western North Icelandic region. The high abundance of the near-surface dwelling *T. quinqueloba* reflects the presence of the Polar Front on the North Icelandic shelf.

**
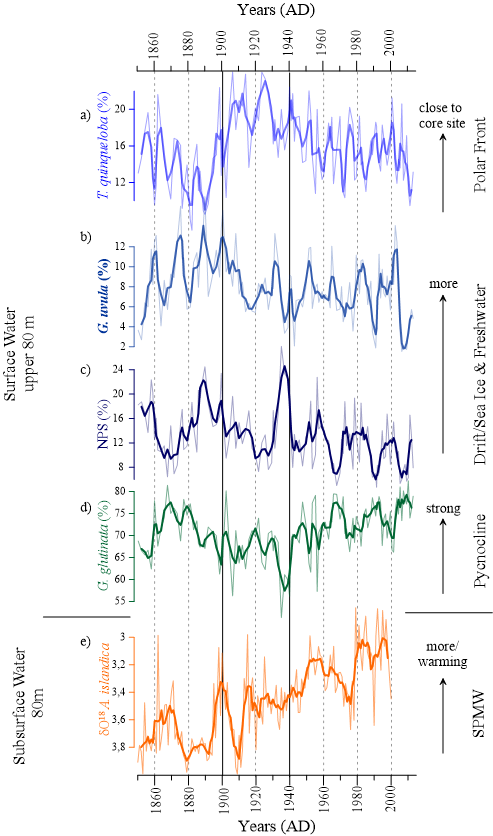
**

**Supplementary Fig. 3: Reconstruction of regional oceanic conditions on the North Icelandic shelf.**

**a to d)** abundance (%) of planktic foraminifera identified in core GS15-198-33 from the western Icelandic shelf. **a)** *Turborotalita quinqueloba*, a subarctic species, thrives in nutrient-rich surface waters associated with oceanic front, i.e. Polar Front^12^. This species blooms particularly in spring in a stratified water column. **b)** *G. uvula* is a cold/subarctic waters, near oceanic front’s and is able to thrive robustly in freshwaters^13-14^ **c)** *Neogloboquadrina pachyderma*, a cold/arctic species prefers nutrient poor surface waters and stable stratified water column^12,16-18^. **d)** *G. glutinata*, a cosmopolitan species that can live in subarctic/subtropical habitats is usually found within the mixed-layer near the chlorophyll maximum^19^. **e)** Stable oxygen isotope (δ^18^O) measured in *Arctic islandica* from ref. 3, a typical North Atlantic mollusc collected from the Grímsey area (see Supplementary Fig. 1) on the eastern North Icelandic shelf. The authors link lower δ^18^O values to a warming of the Subpolar Mode Waters (SPMW) from the Irminger Current that arrive on the North Icelandic shelf.

**Supplementary Note 4:** The surface sea level pressure (SLP) in the region links to large-scale atmospheric circulation patterns, as illustrated by the changes in the winter North Atlantic Oscillation index (NAO)^20^. We performed an empirical orthogonal function (EOF) analysis of the winter season (January-February-March) SLP data for the period 1850-2017 for the northern North Atlantic region (defined by the box 20-70N to 90W-40E), using the HadSLP2 data^21^ (Supplementary Fig. 4). This analysis reveals that the NAO pattern explains 38% of the atmospheric winter variability over the North Atlantic region. During positive (negative) NAO phases, strong southwesterly (northerly) wind anomalies prevail in our study area.

**Supplementary Fig. 4: Patterns of atmospheric variability.** The leading modes of atmospheric winter (December-January-February) sea level pressure variability for the Atlantic sector (90^o^W-40^o^E; 20^o^N-70^o^N) for the period 1850-2017 based on empirical orthogonal function (EOF) analysis of HadSLP2 data^20^. The EOFs are shown as regression maps where the winter sea level pressure data is regressed onto the first four principal components (PCs). The patterns are: a) North Atlantic Oscillation (NAO; 1^st^ PC), b) East Atlantic Pattern (EAP; 2^nd^ PC), c) Scandinavian Pattern (SCP; 3^rd^ PC) and d) East Atlantic Western Russia pattern (EAWR; 4^th^ PC). Units are hPa per standard deviation of the PC. The explained variance in per cent is also shown for each pattern. The first four PCs explain a total of 81% of the atmospheric variability over the North Atlantic region.

**
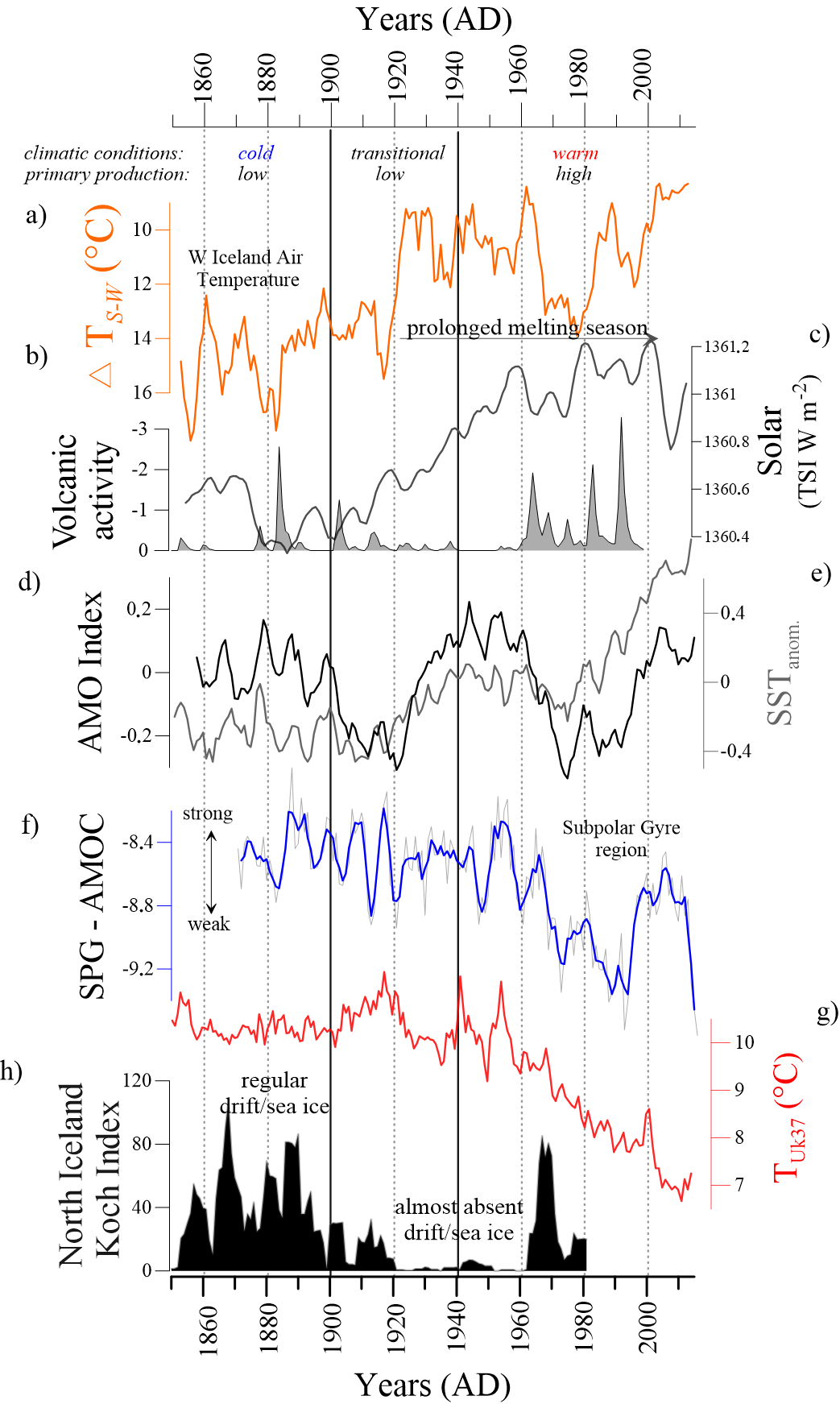
**

**Supplementary Fig. 5: Oceanic changes in the subpolar North Atlantic. a)** Temperature difference between summer (July/August/September) and winter (December/January/February) months calculated from the Stykkishólmur data set. **b)** Volcanic activity as presented in ref. 22. **c)** Solar irradiance as shown in ref. 23. **d)** Atlantic Multidecadal Oscillation (AMO)^24^ Index from the Kaplan SST V2 data, provided by the NOAA ESRL Physical Sciences Division, Boulder, Colorado, USA, from their website at http://www.esrl.noaa.gov/psd. **e)** HadCRUT4 sea surface temperature (SST) anomaly data for the northern hemisphere presented relative to the period 1961-1990^25^. f) Reconstructed Atlantic Meridional Overturning Circulation (AMOC) changes for the Subpolar Gyre region^26^. **g)** U^k^_37_-based SST reconstruction from core GS15-198-33 (this study) according to ref. 27: U^k^_37_=(C_37:2_-C_37:4_)/(C_37:2_+C_37:3_+C_37:4_). The prominent cooling in the reconstructed U^k^_37_ SST’s since the 1960s implies that alkenones were mainly produced in early spring due to the reduced sea ice occurrence in the region. **h)** Drift ice record of historical ice appearance on the North Icelandic shelf^28,29^.

**
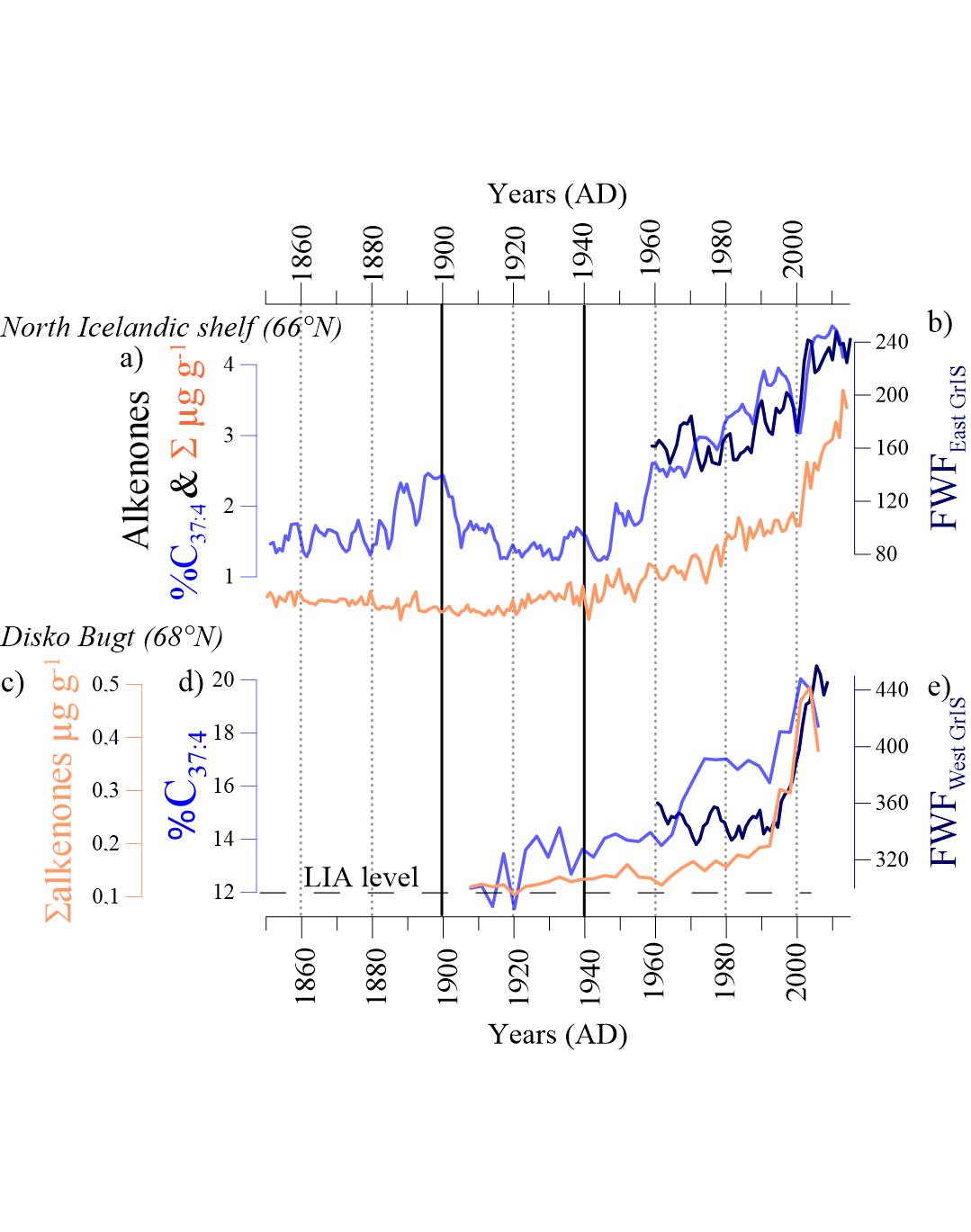
**

**SupplementaryFig. 6. Influence of the Greenland Ice sheet on surface water productivity.** *North Icelandic shelf biomarker:* **a)** %C_37:4_ reflects freshwater occurrence (blue line) and the sum (Σ) of all alkenones predominantly reproduces surface water productivity in spring (this study); **b)** Freshwater Flux (FWF) data as presented in ref. 30 calculated for the east Greenland Ice Sheet area. *Disko Bugt, West Greenland biomarker:* the proxy data of **c)** %C_37:4_ and d) Σ of alkenones have been obtained from the multi core MSM343310^31^ and the **e)** FWF data for the West Greenland Ice Sheet area have been taken from ref. 30.

**Supplementary references**

**1** Giraudeau, J., Jennings, A. E. & Andrews, J. T. Timing and mechanisms of surface and intermediate water circulation changes in the Nordic Seas over the last 10, 000cal years: a view from the North Iceland shelf. *Quaternary Science Reviews* **23**, 2127–2139 (2004).

**2** Schlitzer, R., Ocean Data View, odv.awi.de, 2018.

**3** Reynolds, D. J., et al. Annually resolved North Atlantic marine climate over the last millennium. *Nature Communications* **7**, 1–11, 10.1038/ncomms13502 (2017).

**4** Smith, J. N. Why should we believe 210Pb sediment geochronologies? *Journal of Environmental Radioactivity* **55**, 121–123 (2001).

**5** Appleby, P. G. 2008: Three decades of dating recent sediments by fallout radionuclides: a review. *The Holocene* **18**, 83-93.

**6** Horowitz, H. M., Jacob, D. J., Amos, H. M., Streets, D. G. & Sunderland, E. M. Historical mercury releases from commercial products: Global environmental implications. *Environmental Science & Technology* **48**, 10242-10250 (2014).

**7** Faïn, X., et al. Polar firn air reveals large-scale impact of anthropogenic mercury emissions during the 1970s. *Proceedings of the National Academy of Sciences* **106**, 16114-16119 (2009).

**8** Kaiser, J., Moros, M., Tomczak, M., Dellwig, O., Schulz-Bull, D., & Arz, H. W. (2018). The invasive diatom Pseudosolenia calcar-avis and specific C25 isoprenoid lipids as a sedimentary time marker in the Black Sea. *Geology* **46**, 507-510.

**9** Moros, M., Andersen, T. J., Schulz‐Bull, D., Häusler, K., Bunke, D., Snowball, I., Kotilainen, A., Zillen, L., Jensen, J. B., Kabel, K., Hand, I., Leipe, T., Lougheed, B. C., Wagner, B., & Arz, H. W. (2017) Towards an event stratigraphy for Baltic Sea sediments deposited since AD 1900: approaches and challenges. *Boreas* **46**, 129-142.

**10** Rosell-Melé, A., Jansen, E. & Weinelt, M. Appraisal of a molecular approach to infer variations in surface ocean freshwater inputs into the North Atlantic during the last glacial. *Global Planetary Change* **34**, 143-152 (2002).

**11** Frajka-Williams, E., & Rhines, P. B. Physical controls and interannual variability of the Labrador Sea spring phytoplankton bloom in distinct regions. *Deep Researvh Part I Oceanogr. Res. Pap.* **57**, 541–552 (2010). doi:10.1016/j.dsr.2010.01.003.

**12** Johannesen, T., Jansen, E., Flatøy, A., Ravelo, A.C. The Relationship Between Surface Water Masses, Oceanographic Fronts and Paleoclimatic Proxies in Surface Sediments of the Greenland, Iceland, Norwegian Seas. In: Zahn, R., Kaminski, M. (Eds.), *Carbon Cycling in the Glacial Ocean*. NATO ASI Series I, 17. Springer-Verlag, Berlin/Heidelberg, pp. 61–85 (1994).

**13** Boltovskoy, E., Boltovskoy, D., Correa, N. & Brandini, F. Planktic foraminifera from the southwestern Atlantic (30-60°S), species-specific patterns in the upper 50 m. *Marine Micropaleontology* **28**, 53–72 (1996).

**14** Bergami, C., Capotondi, L., Langone, L., Giglio, F. & Ravaioli, M. Distribution of living planktonic foraminifera in the Ross Sea and the Pacific sector of the Southern Ocean (Antarctica). *Marine Micropaleontology* **73**, 37–48 (2009).

**15** Rasmussen, T. L. & Thomsen, E. Holocene temperature and salinity variability of the Atlantic Water inflow to the Nordic seas. *The Holocene* **20**, 1223-1234 (2010).

**16** Bé, A. H. W. & Tolderlund, D. S. Distribution and ecology of living planktonic foraminifera in surface waters of the Atlantic and Indian Oceans. In: Funnell, B. M. & Riedel, W. R. (eds.): *The Micropaleontology of the Oceans*, 105–149. University Press, Cambridge (1971).

**17** Kohfeld, K. E., Fairbanks, R. G., Smith, S. L., & Walsh, I. D. Neogloboquadrina pachyderma (sinistral coiling) as paleoceanographic tracers in polar oceans: Evidence from Northeast Water Polynya plankton tows, sediment traps, and surface sediments. *Paleoceanography* **11**, 679–699 (1996).

**18** Schiebel, R. & Hemleben, C. Interannual variability of planktic foraminiferal populations and test fluxes in the eastern North Atlantic Ocean (JGOFS). *Deep Sea Research II* **47**, 1809-1852 (2000).

**19** Hemleben, C., Spindler, M. & Anderson, O. R. *Modern Planktonic Foraminifera*. 363 pp. Springer, New York (1989).

**20** Hurrell, J. W. Decadal trends in the North Atlantic Oscillation: Regional temperatures and precipitation. *Science* **269**, 676-679 (1995).

**21** Allan, R. & Ansell, T. A new globally complete monthly historical mean sea level pressure data set (HadSLP2): 1850-2004. *Journal of Climate* **19**, 5816-5842 (2006).

**22** Crowley, T. J. Causes of climate change over the past 1000 years. *Science* **289**, 270-277, 10.1126/science.289.5477.270 (2000).

**23** Coddington, O., Lean, J. L., Pilewskie, P., Snow, M. & Lindholm, D. A solar irradiance climate data record. *Bull. American Meteorological Society* **97**, 1265-1282, 10.1175/BAMS-D-14-00265.1 (2016).

**24** Gray, S. T., Graumlich, L. J., Betancourt, J. L. & Pederson, G. T. A tree-ring based reconstruction of the Atlantic Multidecadal Oscillation since 1567A.D. *Geophysical Research Letters* **31**, L12205 (2004).

**25** Morice, C. P., Kennedy, J. J. Rayner, N. A. & Jones, P. D. Quantifying uncertainties in global and regional temperature change using an ensemble of observational estimates: The HadCRUT4 dataset. *Journal of Geophysical Research* **117**, D08101, 10.1029/2011JD017187 (2012).

**26** Caesar, L., Rahmstorf, S., Robinson, A., Feulner, G., Saba, V. Observed fingerprint of a weakening Atlantic Ocean overturning circulation. *Nature* **556**, 191-196, 10.1038/s41586-018-0006-5 (2018).

**27** Brassell, S. C., Eglinton, G., Marlowe, I. T., Pflaumann, U. & Sarnthein, M. Molecular stratigraphy: a new tool for climatic assessment. *Nature* **320**, 129-133 (1986).

**28** Koch, L. The east Greenland ice. Medd. Grønland 130, 1-374, København (1945).

**29** Wallevik, J. & Sigurjónsson, H. The Koch index. formulation, corrections and extensions. Vedurstofa Íslands Report, VÍ-G98035-ÚR28, Reykjavik, Iceland (1998).

**30** Bamber, J., van den Broeke, M., Ettema, J., Lenaerts, J. & Rignot, E. Recent large increases in freshwater fluxes from Greenland into the North Atlantic. *Geophysical Research Letters* **39**, L19510 (2012).

**31** Moros, M. et al. Surface and sub-surface multi-proxy reconstruction of middle to late Holocene palaeoceanographic changes in Disko Bugt, West Greenland. *Quaternary Science Reviews* **132**, 146-160 (2016).
